# Intracoronary Imaging in Percutaneous Coronary Interventions: The Eye Cannot Appreciate What It Cannot See

**DOI:** 10.1016/j.jscai.2022.100517

**Published:** 2022-11-30

**Authors:** Mahvash Zaman, Muhammad Rashid, Mamas A. Mamas

**Affiliations:** a Keele Cardiovascular Research Group, School of Medicine, Keele University, Stoke-on-Trent, United Kingdom; b Department of Cardiology, Wythenshawe Hospital, Manchester Foundation Trust, Manchester, United Kingdom; c Department of Cardiology, Freeman Hospital, Newcastle, United Kingdom; d Department of Academic Cardiology, Royal Stoke University Hospital, Stoke-on-Trent, United Kingdom

**Keywords:** angiography, intravascular ultrasound, optical coherence tomography

The use of intracoronary imaging (ICI) in the form of intravascular ultrasound (IVUS) and optical coherence tomography (OCT) is associated with improved periprocedural and long-term clinical outcomes in patients undergoing percutaneous coronary intervention (PCI). The advantage of ICI becomes even greater when treating patients with complex lesion morphology or acute coronary syndrome.^1^ ICI allows real-time assessment of lesion characteristics and plaque morphology, collection of detailed information about vessel anatomy and size, and assessment of stent apposition and expansion. International guidelines recommend the use of ICI in patients undergoing PCI, with North American and European guidelines providing level IIa recommendations based on large-scale randomized controlled trial (RCT) data.^2^^,^^3^

The use of both IVUS and OCT lead to superior outcomes compared with angiography-guided PCI; however, each has inherent strengths and limitations. The mechanism of IVUS leading to superior outcomes is believed to be through accurate vessel sizing with a higher frequency of stent post-dilatation, leading to larger minimal stent areas and lower rates of geographic miss. OCT produces high-resolution images with automated quantification of vessel lumen dimensions, increased sensitivity in identifying plaque morphology, and post-PCI findings, such as edge dissection. Recent studies providing dedicated algorithms for the use of OCT according to the external elastic membrane have shown noninferiority to IVUS^4^^,^^5^; however, large-scale studies on the head-to-head comparison of IVUS and OCT are still lacking.

In the current issue of *JSCAI*, Shariff et al^6^ present the findings of an updated network meta-analysis of 14 RCTs published over the previous decade, including 6816 patients who underwent drug-eluting stent implantation with IVUS, OCT, or angiography guidance alone. Both OCT and angiography were compared with IVUS. The analysis is well conducted and adds to the existing literature, demonstrating that angiography-guided PCI compared with IVUS was associated with higher odds of major adverse cardiovascular events (MACE) (odds ratio [OR], 1.64; 95% CI, 1.30-2.07), target vessel revascularization (TVR) (OR, 1.61; 95% CI, 1.21-2.14), and cardiovascular mortality (OR, 1.97; 95% CI, 1.25-3.11). There was no difference in the odds of myocardial infarction (OR, 1.18; 95% CI, 0.81-1.73) or all-cause mortality (OR, 0.97; 95% CI, 0.70-1.35).

When comparing outcomes associated with the use of IVUS and OCT, the odds of MACE (OR, 1.31; 95% CI, 0.81-2.11), myocardial infarction (OR, 0.93; 95% CI, 0.42-2.06), TVR (OR, 1.33; 95% CI, 0.75-2.37), all-cause mortality (OR, 2.55; 95% CI, 0.74-8.81), and cardiovascular mortality (OR, 1.19; 95% CI, 0.20-7.20) were similar among participants who underwent OCT-guided PCI when compared with participants who underwent IVUS-guided PCI. This finding is consistent with those in previous studies,^7^ in which IVUS and OCT have been shown to perform comparably with respect to their effect on the procedural result in addition to midterm clinical outcomes of PCI.^5^

Nevertheless, the analysis has several limitations that are common to publication-level meta-analyses. The current analysis provides no information on lesion characteristics or the patient groups that would benefit from ICI. Recent data from the British Cardiovascular Intervention Society suggest that the main prognostic benefit of ICI is derived from the use of imaging in cases with a European Association of Percutaneous Cardiovascular Interventions imaging-recommended indication, including stent thrombosis, in-stent restenosis, renal failure, bioresorbable vascular scaffolds, a stent length of >60.0 mm, acute coronary syndrome indications, chronic total occlusion, and left main stem intervention.^1^ Furthermore, this analysis does not inform whether studies achieved guideline-recommended imaging criteria for optimal PCI. In the ULTIMATE trial,^8^ the benefit of IVUS was only observed in those that achieved imaging criteria for optimal PCI, namely a minimal lumen area in the stented segment of >5.0 mm^2^ or 90% of the distal reference lumen minimal stent area, a plaque burden 5.0-mm proximal or distal to the stent edge of <50%, and no edge dissection involving media with a length of >3.0 mm. Patients in the ICI arm in whom criteria were not achieved had similar outcomes to those in the angiography-guided arm.

So how does the current analysis inform our choice of ICI? The findings of the current study reaffirm what we know about ICI and provide further evidence to support the requirement to use ICI to achieve optimal results with drug-eluting stent placement. Nevertheless, given the lack of robust comparative data, it cannot answer whether the use of IVUS or OCT is associated with better outcomes. The optimal choice of ICI should depend on both the indication for imaging and operator experience/preference. Recently, a survey performed by the European Association of Percutaneous Cardiovascular Interventions and the Japanese Association of Cardiovascular Interventions and Therapeutics (CVIT)^9^ revealed that 95.5% of operators had experience with IVUS, whereas 69.8% of operators had experience with OCT. Despite the difference in experience, most respondents perceived equipoise regarding the choice between IVUS and OCT, and >50% believed that patient characteristics and coronary anatomy should guide the mode of ICI.

Despite the growing evidence base derived from RCTs around the positive effect of ICI on clinical outcomes and several international guideline recommendations, the use of ICI remains low globally, with reports of 4.8% use in PCI in the United States and <18% in all-comer PCI in the United Kingdom.^1^^,^^10^ As a community of interventional cardiologists, we need to explore factors that have served as barriers to broader adoption. Possible reasons for the underuse of ICI may relate to a lack of structured learning/training during interventional training programs. Perceived time constraints and costs are other reasons that may prevent the use of ICI; however, with frequent use, operators can expect to become more efficient with using ICI, and long-term cost benefits have been demonstrated.

**Figure 1:**
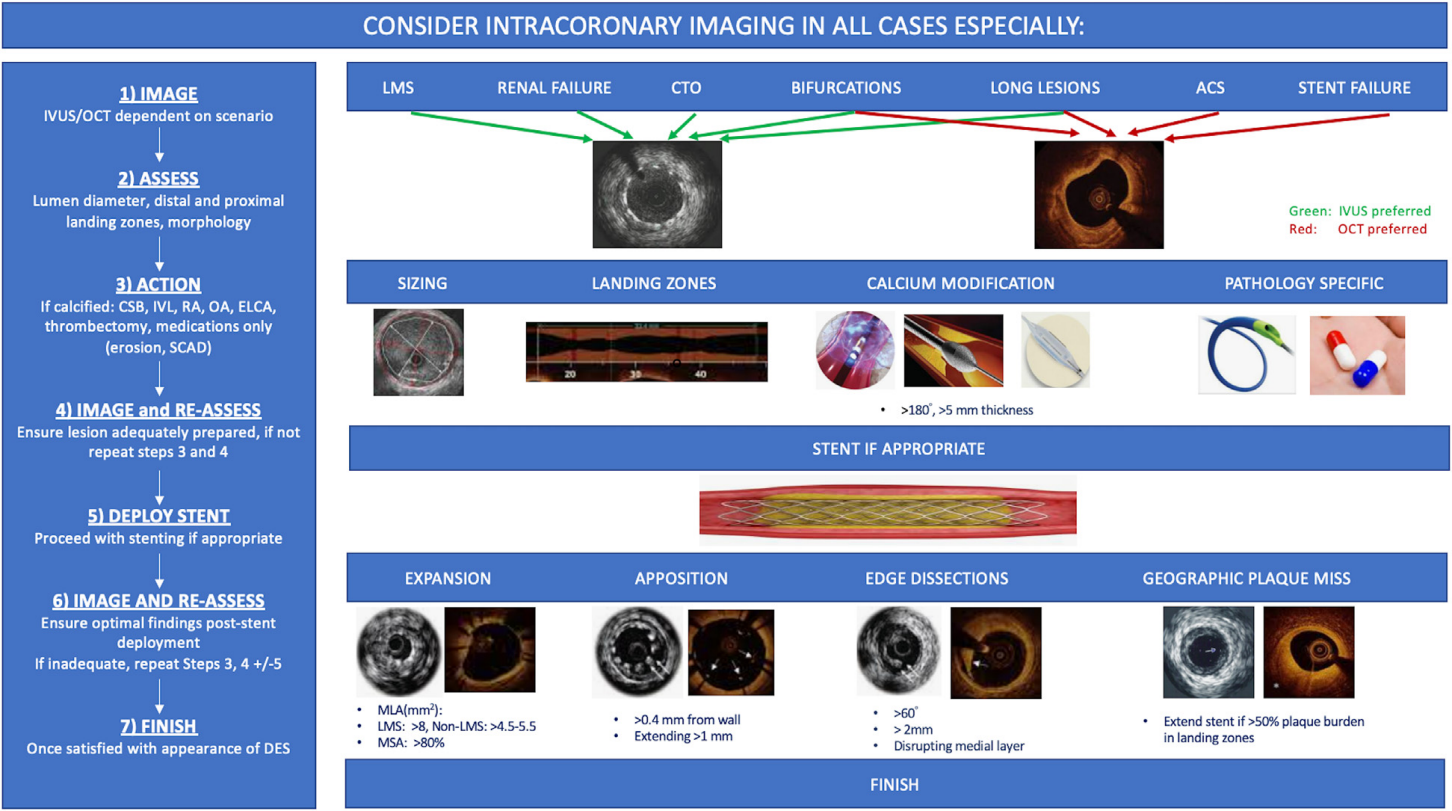
**Algorithm for the use of intracoronary imaging.** ACS, acute coronary syndrome; CSB, cutting/scoring balloon; CTO, chronic total occlusion; DES, drug-eluting stent; ELCA, excimer laser coronary angioplasty; IVL, intravascular lithotripsy; IVUS, intravascular ultrasound; LMS, left main stem; MLA, minimal luminal area; MSA, minimal stent area; OA, orbital atherectomy; OCT, optical coherence tomography; RA, rotational atherectomy; SCAD, spontaneous coronary artery dissection.

So, where does this leave us? The question should not be which ICI modality to use but rather why is ICI not being used more widely. Second, when using ICI, we should be aiming for an optimal PCI defined by validated imaging criteria. Merely passing an ICI catheter down a coronary artery will not improve clinical outcomes (Figure 1). Third, there are few interventions in PCI that can reduce MACE and TVR by close to 40% and cardiovascular mortality by close to 50%—we owe it to our patients to overcome any personal barriers that we may have in using this technology. Finally, the eye cannot appreciate what it cannot see—being blind should no longer be an excuse for not performing optimal PCI.
